# Parabens as Urinary Biomarkers of Exposure in Humans

**DOI:** 10.1289/ehp.9413

**Published:** 2006-08-29

**Authors:** Xiaoyun Ye, Amber M. Bishop, John A. Reidy, Larry L. Needham, Antonia M. Calafat

**Affiliations:** Division of Laboratory Sciences, National Center for Environmental Health, Centers for Disease Control and Prevention, Atlanta, Georgia, USA

**Keywords:** biomonitoring, conjugate, ethylparaben, metabolism, methylparaben, butylparaben, *n*-propylparaben, *p*-hydroxybenzoic acid esters, urine

## Abstract

**Background:**

Parabens appear frequently as antimicrobial preservatives in cosmetic products, in pharmaceuticals, and in food and beverage processing. *In vivo* and *in vitro* studies have revealed weak estrogenic activity of some parabens. Widespread use has raised concerns about the potential human health risks associated with paraben exposure.

**Objectives:**

Assessing human exposure to parabens usually involves measuring in urine the conjugated or free species of parabens or their metabolites. In animals, parabens are mostly hydrolyzed to *p*-hydroxybenzoic acid and excreted in the urine as conjugates. Still, monitoring urinary concentrations of *p*-hydroxybenzoic acid is not necessarily the best way to assess exposure to parabens. *p*-Hydroxybenzoic acid is a nonspecific biomarker, and the varying estrogenic bioactivities of parabens require specific biomarkers. Therefore, we evaluated the use of free and conjugated parent parabens as new biomarkers for human exposure to these compounds.

**Results:**

We measured the urinary concentrations of methyl, ethyl, *n-*propyl, butyl (*n-* and *iso*-), and benzyl parabens in a demographically diverse group of 100 anonymous adults. We detected methyl and *n-*propyl parabens at the highest median concentrations (43.9 ng/mL and 9.05 ng/mL, respectively) in nearly all (> 96%) of the samples. We also detected other parabens in more than half of the samples (ethyl, 58%; butyl, 69%). Most important, however, we found that parabens in urine appear predominantly in their conjugated forms.

**Conclusions:**

The results, demonstrating the presence of urinary conjugates of parabens in humans, suggest that such conjugated parabens could be used as exposure biomarkers. Additionally, the fact that conjugates appear to be the main urinary products of parabens may be important for risk assessment.

Parabens are esters of *p-*hydroxybenzoic acid, widely used as antimicrobial preservatives—especially against molds and yeast—in cosmetic products and pharmaceuticals and in food and beverage processing (Elder 1984; Orth 1980; Weber 1993). Cosmetics manufacturers and food processers use methyl and propyl parabens most extensively (Elder 1984; Jackson 1992). Parabens are popular because of their low toxicity and cost, broad inertness, and worldwide regulatory acceptance (Soni et al. 2005). Parabens are not mutagenic (Elder 1984), but their potential estrogenic activity—although many orders of magnitude lower than that of estrogen (Golden et al. 2005; Routledge et al. 1998)— has raised some concerns about their toxicity. *In vitro* data suggest that parabens demonstrate weakly estrogen activities in yeast-based assays (Miller et al. 2001; Nishihara et al. 2000; Routledge et al. 1998; Vinggaard et al. 2000), induce the growth of MCF-7 human breast cancer cells, and influence the expression of estrogen-dependent genes (Byford et al. 2002; Darbre et al. 2002, 2003; Okubo et al. 2001). On the other hand, increased uterine weights have been reported in immature mice after exposure to *n-*butyl, *iso*-butyl, and benzyl parabens (Darbre et al. 2002). Decreased excretion of testosterone and alterations in the male reproductive tract were observed in male rodents after exposure to butyl and propyl parabens (Oishi 2001, 2002a, 2002b) but not to methyl and ethyl parabens (Oishi 2004). Ethyl, propyl, and butyl parabens evoked estrogenic responses in sexually immature rainbow trout, although the estrogenic potency of ethyl paraben was weaker than that of propyl and butyl paraben (Pedersen et al. 2000).

Data on human exposure to parabens are limited (Darbre et al. 2004; Makino 2003; National Toxicology Program 2004), and the toxic effects of parabens in humans are mostly unknown. The detection (but not concentration ranges) of methyl paraben in cord blood and breast milk has been reported (Makino 2003). A study on the presence of parabens in human breast tumors (Darbre et al. 2004) triggered debate over the use of parabens in cosmetics, particularly in underarm deodorants and antiperspirants, which may increase the incidence of breast cancer (Darbre 2001, 2004, 2006; Elder 1984; Golden et al. 2005; Harvey 2003, 2004; Harvey and Darbre 2004; Harvey and Everett 2004).

Such findings, involving as they do the estrogenicity of parabens in animals and the presence of parabens in human breast tissue, have raised questions about the safety of widespread paraben use, and these questions prompted the Cosmetic Ingredient Review program (established in 1976 by the Cosmetics, Toiletry, and Fragrance Association), with the support of the Food and Drug Administration (FDA) and the Consumer Federation of America, to reevaluate paraben safety (Bergfeld et al. 2005). Furthermore, the National Institute of Environmental Health Sciences nominated butyl paraben for toxicological characterization, including reproductive toxicity studies (National Toxicology Program 2004).

After intraduodenal or intravenous administration (2 mg/kg) in rats, parabens are mainly hydrolyzed to *p*-hydroxybenzoic acid, which can then be conjugated with glycine, glucuronide, and sulfate and excreted in urine (Cashman and Warshaw 2005; Kiwada et al. 1979, 1980; Soni et al. 2005). In rabbits, after oral administration of methyl-, ethyl-, propyl-, and butyl parabens at doses of 0.4 and 0.8 g/kg, a small percentage (0.2–0.9%) of the unchanged paraben was excreted in the urine (Tsukamoto and Terade 1960, 1962, 1964).

Although human toxicokinetic data are limited, some reports state that after oral administration of propyl paraben (2 g for 5 days) to a human volunteer, only 17.4% of the administered dose was recovered as *p-*hydroxybenzoic acid and its conjugates in urine (Sabalitschka 1954). Another report concerning six preterm infants who were injected intramuscularly with a paraben*-*containing gentamicin formulation showed that the urinary excretion of methyl paraben could range from 13.2 to 88.1% (Hindmarsh et al. 1983). Most of the methyl paraben was excreted in urine in its conjugated form.

Measuring *p*-hydroxybenzoic acid or its conjugates in urine may not represent the optimal approach for assessing human exposure to parabens. *p*-Hydroxybenzoic acid is a nonspecific metabolite of all parabens, and individual parabens are known to have quite different estrogenic bioactivities (Golden et al. 2005). In the present study, we evaluated the use of the free and conjugated species of the parent parabens as new biomarkers for human exposure to these compounds.

## Materials and Methods

Methyl, ethyl, *n-*propyl, *n-*butyl, and benzyl paraben, 4-methylumbelliferyl glucuronide, 4-methylumbelliferyl sulfate, and β-glucuronidase/sulfatase (*Helix pomatia*, H1) were purchased from Sigma Chemical Co. (St. Louis, MO). ^13^C_4_-4-Methylumbelliferone was purchased from Cambridge Isotope Laboratories, Inc. (Andover, MA). d_4_-Methyl paraben was purchased from C/D/N Isotopes Inc. (Quebec, Canada). d_4_-Ethyl, d_4_-*n-*propyl, and d_4_-*n-*butyl parabens were obtained from CanSyn Chem Corp. (Toronto, Canada). Because d_4_-benzyl parabens was not available, d_4_-*n-*butyl paraben was used as the internal standard for benzyl paraben. The urine samples analyzed for this study were collected anonymously from a demographically diverse group of 100 U.S. male and female adults with no known exposure to parabens. The samples were collected from 2003 to 2005 at different times throughout the day—not necessarily first morning voids. The Centers for Disease Control and Prevention Human Subjects Institutional Review Board reviewed and approved the study protocol. A waiver of informed consent was requested under the Code of Federal Regulations dealing with human subjects [45 CFR 46.116(d)].

We measured the total concentrations of methyl, ethyl, *n-*propyl, butyl (*n-* and *iso*-), and benzyl parabens with a modification of a method for measuring other environmental phenols in urine (Ye et al. 2005a) using online solid-phase extraction high-performance liquid chromatography–tandem mass spectrometry (SPE-HPLC-MS/MS) (Ye et al. 2006). Briefly, 100 μL urine was spiked with 50 μL internal standard solution, 50 μL enzyme solution, and, to monitor the completion of the deconjugation reaction, 10 μL 4-methylumbelliferyl glucuronide/4-methylumbelliferyl sulfate/^13^C_4_-4-methylumbelliferone standard solution (Ye et al. 2006). Samples were incubated for 4 hr at 37°C and then acidified with 0.1 M formic acid. The online SPE-HPLC-MS/MS system consisted of several Agilent 1100 modules (Agilent Technologies, Wilmington, DE)—two binary HPLC pumps, an autosampler with a 900-μL injection loop, and one column compartment with a 10-port switching valve, and an API 4000 triple quadrupole mass spectrometer (Applied Biosystems, Foster City, CA) equipped with an atmospheric pressure chemical ionization interface. The procedure for extracting the deconjugated parabens from the urine involved concurrent online SPE-HPLC operation with peak focusing. While the autosampler and one of the HPLC pumps were used for the SPE cleanup of one sample, the 10-port switching valve, the second HPLC pump, and the mass spectrometer were used to collect data from the previous sample (Ye et al. 2006). The limits of detection (LODs), calculated as 3S_0_, where S_0_ is the standard deviation as the concentration approaches zero (Taylor 1987), were 0.13 ng/mL (methyl paraben), 0.18 ng/mL (*n-*propyl paraben), and 0.1 ng/mL (ethyl, butyl, and benzyl parabens) (Ye et al. 2006).

To determine the concentrations of the free species (C_free_), we followed the above procedure without the enzymatic hydrolysis treatment. We used β-glucuronidase (*Escherichia coli*-K12; Roche Applied Science, Penzberg, Germany) to selectively deconjugate glucuronide metabolites and determine the concentrations of free plus glucuronidated species (C_free+glu_). Similarly, we used β-glucuronidase/sulfatase (*H. pomatia*, 463 000U/g solid) to deconjugate both glucuronide and sulfate metabolites and to determine the concentrations of free plus glucuronidated plus sulfated species (C_free+glu+sul_). We calculated the concentration of glucuronide conjugates by subtracting C_free_ from C_glu+free_. Similarly, we calculated the concentration of sulfate metabolites by subtracting C_free+glu+sul_ from C_free+glu_ (Ye et al. 2005b).

To ensure accuracy and precision of the data, we analyzed quality control (QC) materials along with the samples. Low-concentration (QCL, 2–5 ng/mL) and high-concentration (QCH, 10–50 ng/mL) QC materials were prepared from a base urine pool obtained from multiple anonymous donors (Ye et al. 2006). The materials were dispensed in 1-mL aliquots and stored at −70°C. Each QC material was characterized to define the mean and the 95% and 99% control limits of parabens concentrations using a minimum of 40 repeated measurements during a 2-week period.

We performed the statistical analyses using the Statistical Analysis System (SAS Institute, Cary, NC) software. For concentrations below the LOD, we used a value equal to the LOD divided by the square root of 2 (Hornung and Reed 1990). Statistical significance was set at *p* < 0.05.

## Results and Discussion

We measured total (i.e., free plus glucuronidated and sulfated conjugates) concentrations of five parabens in 100 human urine samples collected between 2003 and 2005 from 100 adult anonymous volunteers with no known occupational exposure to these compounds. The median concentrations, selected percentiles, and frequency of detection of each paraben are all shown in Table 1.

**Figure 1:**
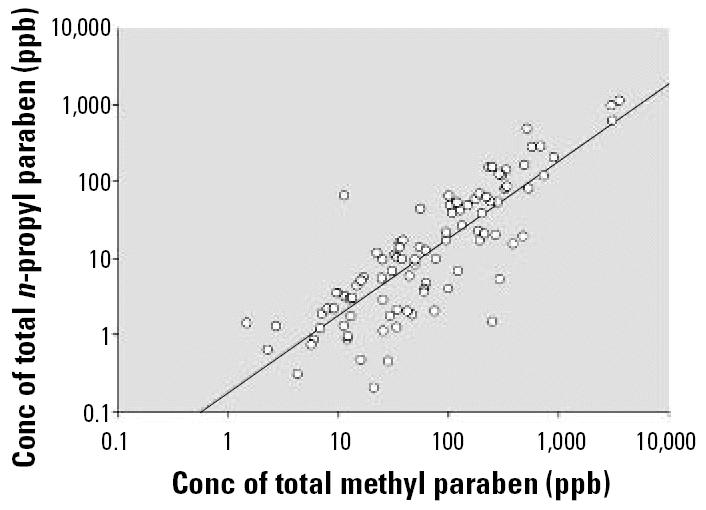
Correlation analyses of total urinary concentrations of methyl and *n-*propyl parabens limited to samples with detectable levels of each paraben (*n* = 93; *r* = 0.92; *p* < 0.0001). Conc, concentration. When all samples were included in the analysis, the *r-* and *p*-values were very similar.

Methyl and *n-*propyl paraben were detected in almost all of the samples (99% and 96%, respectively). Two other parabens were detected in more than half of the samples (ethyl, 58%; butyl, 69%); benzyl paraben was detected less frequently (39%) (Table 1). Median urinary concentrations were highest for methyl (43.9 ng/mL) and *n-*propyl (9.1 ng/mL) parabens. Methyl paraben was also the most abundant (mean value of 12.8 ng/g) among 6 parabens tested in human breast tumors (Darbre et al. 2004). The higher frequency of detection and median urinary concentrations of methyl and *n-*propyl parabens could be related to *a*) the fact that these are the most widely used parabens in cosmetics and food processing (Jackson 1992), *b*) differences in the absorption, distribution, metabolism, and excretion of the various parabens, or *c*) a combination of these factors. Furthermore, the high correlation (Pearson correlation coefficient *r =* 0.92, *p* < 0.0001) between total urinary concentrations of methyl and *n-*propyl parabens (Figure 1) suggests that human exposures to methyl and *n-*propyl parabens most likely share common sources.

Differences in metabolic profiles have been found depending on the exposure route (Bando et al. 1997; Daston 2005; Harvey 2005; Soni et al. 2005). After oral administration, parabens are most likely hydrolyzed by nonspecific esterases, widely distributed in the body, including the gut (Daston 2005). After dermal exposure, parabens can also be hydrolyzed by esterases present in human skin and subcutaneous fat tissue (Lobemeier et al. 1996). The presence of unchanged parabens in breast tumor tissues (Darbre et al. 2004) suggested, however, that at least a fraction of the parabens can be absorbed without hydrolysis. The high frequency of detection of some parabens in our study confirms that, after exposure, unhydrolyzed parabens may be excreted in urine.

**Figure 2:**
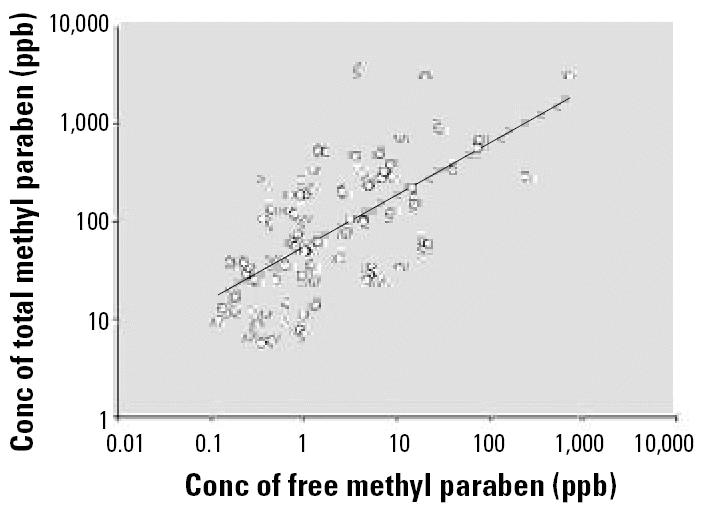
Correlation analyses of total versus free concentrations of methyl paraben for samples with detectable values (*n* = 83; *r* = 0.67; *p* < 0.0001). Conc, concentration. When all samples (*n* = 100) were included in the analysis, we obtained very similar *r-* and *p*-values.

The median concentrations of free methyl, ethyl, and *n-*propyl parabens were lower than their corresponding median total concentrations (Table 1). For methyl and *n-*propyl parabens, the total concentrations were about 50- and 10-fold higher, respectively, than the median concentrations of free species. This suggests that these parabens are mostly excreted in urine as conjugates (Table 1). Moreover, the correlations between the concentrations of free and total (free plus conjugated) species of methyl and *n-*propyl parabens were highly significant (*p* < 0.0001 and *p* = 0.0096, respectively; Figure 2). In other words, high concentrations of total methyl- or *n-*propyl parabens tended to be related to high concentrations of the corresponding free paraben. These findings suggest that parabens, like many xenobiotics, undergo phase II biotransformations to produce glucuronide or sulfate conjugates with increased water solubility that are more amenable to urinary excretion than are the free species (de Wildt et al. 1999; Wang and James 2006). If the biologically active compound were the free species (van den Berg et al. 2003), urinary excretion of the conjugated species would reduce the bioavailable concentration of the free species for target organs, thus minimizing the potential adverse effects related to parabens exposure.

**Figure 3:**
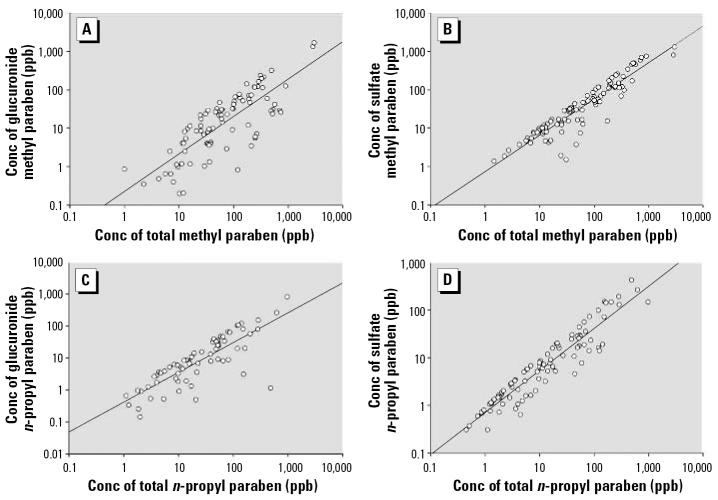
Correlation analyses of total versus conjugated concentrations of (*A*) glucuronidated and (*B*) sulfated methyl paraben, and (*C*) glucuronidated and (*D*) sulfated *n*-propyl paraben. Concentrations < LOD were excluded in the graphical representations (*n* varied from 65 to 97, *r*-values ranged from 0.78 to 0.93, *p* < 0.0001). Conc, concentration. When all 100 samples were included in the analyses, the *r-* and *p*-values were very similar.

We also examined the distribution of glucuronide and sulfate conjugates of parabens (Table 2). The sulfate conjugate represented 67% (methyl paraben) and 55% (*n-*propyl paraben) of the total amount of each paraben excreted in urine. The percentage of the glucuronide conjugate was 28% for methyl paraben, and 43% for *n-*propyl paraben; only a small amount of these parabens was excreted in free form (5% for methyl and 2% for *n-*propyl). The urinary concentrations of both glucuronide and sulfate conjugates correlated well with the concentrations of the total species (*r* ranged from 0.78 to 0.93) (Figure 3). The concentration of conjugated species increased with the concentration of total species, even at the highest concentrations of total species. These findings are in agreement with data on exposure of humans to phthalates, another class of environmental chemicals (Silva et al. 2006). The findings also suggest that saturation or inhibition of the enzymes catalyzing the glucuronidation or sulfatation reactions did not occur at environmental exposure levels.

In conclusion, our preliminary data suggest that parabens can be present in urine, mostly in their conjugated form. To our knowledge, this is the first comprehensive report on the urinary concentrations of individual parabens and their conjugates in humans. The high frequency of detection for methyl, ethyl, *n-*propyl, and butyl parabens suggests that parabens and their conjugates could be valid biomarkers to assess human exposures to these compounds. Additional information, including a better understanding of the metabolism of parabens in humans, is needed to link these biomarker measurements to exposure and to internal dose.

## Figures and Tables

**Table 1:** Total (free plus conjugated) and free urinary concentrations of parabens (ng/mL) at selected percentiles, and frequency of detection in adults (*n* = 100).

		Percentile					
Compound	Frequency of detection (%)	5th	25th	50th	75th	90th	95th
Methyl paraben, total	99	4.2	14.6	43.9	180	412	680
Methyl paraben, free	75	< LOD	0.1	0.8	4.7	15.0	27.8
Ethyl paraben, total	58	< LOD	< LOD	1.0	6.9	25.1	47.5
Ethyl paraben, free	22	< LOD	< LOD	< LOD	< LOD	0.5	1.5
*n*-Propyl paraben, total	96	0.2	1.9	9.1	49.2	144	279
*n-*Propyl paraben, free	37	< LOD	< LOD	< LOD	0.4	1.8	3.4
Butyl paraben, total	69	< LOD	< LOD	0.5	3.3	14.5	29.5
Butyl paraben, free	17	< LOD	< LOD	< LOD	< LOD	0.2	0.3
Benzyl paraben, total	39	< LOD	< LOD	< LOD	0.2	0.4	0.5
Benzyl paraben, free	0	< LOD	< LOD	< LOD	< LOD	< LOD	< LOD

The LODs were 0.13 ng/mL (methyl paraben), 0.18 ng/mL (*n-*propyl paraben), and 0.10 ng/mL (ethyl, butyl, and benzyl parabens). For the statistical calculations, concentrations < LOD were imputed a value of LOD divided by the square root of 2.

**Table 2:** Urinary concentrations of the free, glucuronidated, and sulfated conjugates of methyl and *n-*propyl parabens in adults (*n* = 100).

Compound	Frequency of detection (%)	Median (ng/mL)	Range (ng/mL)	Percentage of total amount
Methyl paraben, free	75	0.8	< LOD–717	5
Methyl paraben, glucuronide	85	9.7	< LOD–1,670	28
Methyl paraben, sulfate	96	29.9	< LOD–1,300	67
*n-*Propyl paraben, free	37	< LOD	< LOD–95.0	2
*n-*Propyl paraben, glucuronide	64	3.2	< LOD–820	43
*n-*Propyl paraben, sulfate	83	5.2	< LOD–424	55

The LODs were 0.13 ng/mL (methyl paraben) and 0.18 ng/mL (*n-*propyl paraben). For the statistical calculations, concentrations < LOD were imputed a value of LOD divided by the square root of 2.
